# Autophagy Is Involved in the Viability of Overexpressing Thioredoxin *o*1 Tobacco BY-2 Cells under Oxidative Conditions

**DOI:** 10.3390/antiox10121884

**Published:** 2021-11-25

**Authors:** Sabrina De Brasi-Velasco, Omar López-Vidal, María Carmen Martí, Ana Ortiz-Espín, Francisca Sevilla, Ana Jiménez

**Affiliations:** Department of Stress Biology and Plant Pathology, CEBAS-CSIC, 30100 Murcia, Spain; sdebrasi@cebas.csic.es (S.D.B.-V.); omalopez17@yahoo.com (O.L.-V.); mcmarti@cebas.csic.es (M.C.M.); anamaria.ortizespin@gmail.com (A.O.-E.); fsevilla@cebas.csic.es (F.S.)

**Keywords:** ATG4, autophagy flux, cell death, hydrogen peroxide, protein interaction, redox regulation

## Abstract

Autophagy is an essential process for the degradation of non-useful components, although the mechanism involved in its regulation is less known in plants than in animal systems. Redox regulation of autophagy components is emerging as a possible key mechanism with thioredoxins (TRXs) proposed as involved candidates. In this work, using overexpressing PsTRX*o*1 tobacco cells (OEX), which present higher viability than non-overexpressing cells after H_2_O_2_ treatment, we examine the functional interaction of autophagy and PsTRX*o*1 in a collaborative response. OEX cells present higher gene expression of the ATG (Autophagy related) marker ATG4 and higher protein content of ATG4, ATG8, and lipidated ATG8 as well as higher ATG4 activity than control cells, supporting the involvement of autophagy in their response to H_2_O_2_. In this oxidative situation, autophagy occurs in OEX cells as is evident from an accumulation of autolysosomes and ATG8 immunolocalization when the E-64d autophagy inhibitor is used. Interestingly, cell viability decreases in the presence of the inhibitor, pointing to autophagy as being involved in cell survival. The in vitro interaction of ATG4 and PsTRX*o*1 proteins is confirmed by dot-blot and co-immunoprecipitation assays as well as the redox regulation of ATG4 activity by PsTRX*o*1. These findings extend the role of TRXs in mediating the redox regulation of the autophagy process in plant cells.

## 1. Introduction

Autophagy is a degradative process in which damaged or non-useful cellular components, including proteins, aggregates, and whole organelles, are degraded in a lytic cell compartment for recycling or elimination. This process has been observed as contributing to antioxidant defenses [1,2] and, similar to that described for other eucaryotes, it is activated and regulated by cells to cope with biotic and abiotic stress [3]. While this process has been less studied in plants than in animal systems, basal autophagy seems to be essential for growth and development in both cases [4,5]. More than thirty proteins participate in the autophagy process, which is regulated by different signaling and stress detection pathways, nutrient availability, metabolic activity, and environmental changes [2,6,7]. Three types of autophagy have been described in animals: Microautophagy, autophagy mediated by chaperones, and the most studied, macroautophagy [8]. In plants, micro- and macroautophagy have been described, as has mega-autophagy, an extreme process occurring in programmed cell death during development and pathogen attack, when the tonoplast releases hydrolases into the cytoplasm after its breakdown [9]. During macroautophagy, specialized double-membrane vesicles, termed autophagosomes, deliver the cytoplasmic content to lytic compartments such as lysosomes or vacuoles in animal and plant systems, respectively, for their hydrolytic degradation [10]. In plants, autophagosomes are produced de novo and the external membrane fuses to the tonoplast, delivering the content as a single-membrane autophagic vesicle in the lumen of the vacuole [3]. The autophagosomes can acquire hydrolytic enzymes from the vacuole to generate vesicles similar to the so-called autolysosomes in animals [5]. In both animal and plant cells, autophagy involves several conserved specific proteins termed ATG (Autophagy related), forming complexes and ultimately generating the double-membrane autophagosome [3]. Among core ATG proteins, ATG8 plays a key role and is associated with the membrane of the autophagosome by covalent binding to phosphatidylethanolamine (PE) at a glycine residue exposed at the C terminus of ATG8. Prior to the conjugation of ATG8 and PE, the cysteine protease ATG4 processes the C terminus of nascent ATG8 to expose the conserved glycine residue (priming activity). Then ATG8 is activated via the ubiquitin-conjugating E1-like enzyme, ATG7, and the E2-like enzyme, ATG3, to finally conjugate to PE. ATG4 participates again with a deconjugating activity that cleaves the PE residue from ATG8, thus allowing ATG8 recycling [11]. In silico analysis of 18 available plant genomes identified a large number of ATG4 and ATG8 genes that are conserved among plant lineages. While *Chlamydomonas reinhardtii* presents one ATG4 gene and one ATG8 gene, *Arabidopsis thaliana* presents two ATG4 and nine ATG8 and *Nicotiana tabacum* has one ATG4 and five ATG8 [12,13]. 

Thioredoxins are small oxidoreductase proteins involved in the redox regulation of specific target proteins, which they reduce from their oxidized forms, being able to regulate their structure and function [14,15,16,17]. Oxidative stress inhibits the protein degradation pathway and autolysosome maturation, provoking apoptosis in neuroblastoma cells, and it has been established that the thioredoxin system plays a central role in inhibiting apoptosis in human cancer cells [18]. Moreover, a more recent role for TRX and thioredoxin reductase (TR) in the initiation of autophagy has been suggested. It is known that this system plays a role in early protective autophagy while its deficiency promotes apoptosis, a situation that can be avoided using N-acetylcysteine, promoting cell viability [19]. TR is also involved in the stability of a key component in the autophagy process: Target of Rapamycin (TOR) kinase [20]. Regarding, ATG4 is the only ATG that has been shown to be redox regulated. Specifically, redox regulation of ATG4 protease by thioredoxin has been demonstrated in animal systems, Chlamydomonas and *Sacharomyces cerevisiae* [21,22], but not in plants. Among TRXs, PsTRX*o*1 is located in the plant mitochondria and nucleus [23] and is involved in the response to oxidative stress induced under adverse situations, such as salinity, high temperature, drought, and H_2_O_2_ treatment, exerting its function on specific target proteins in both compartments [17,24,25,26,27]. We have previously described the involvement of the protein PsTRX*o*1 in the response to oxidative stress induced by H_2_O_2_ in *Nicotiana tabacum* L. cv. Bright Yellow 2 (BY-2) cell cultures overexpressing (OEX) this protein. Interestingly, OEX lines maintained a higher level of cellular viability than non-overexpressing cells, and in this scenario, delayed cell death, the antioxidant system and redox homeostasis collaborate with TRX*o*1 in the resistance to H_2_O_2_ [26,28]. However, it is unclear whether the autophagy process also participates in this increase in cell viability and, if this is so, whether PsTRX*o*1 also plays a part as it has been described in animal systems. The role of autophagy in promoting plant viability during abiotic stress has been demonstrated [29], although this process is also involved in promoting programmed cell death (PCD) in barley during microspore embryogenesis [30]. This would indicate that, as has been shown during biotic stress, the dual function of autophagy depends on the type of stress, plant organ, and developmental stage [31]. In this work, we show that the overexpression of PsTRX*o*1 participates in the response of TBY-2 cells to H_2_O_2_ treatment favoring an autophagy process, which plays a role in facilitating the survival of OEX cells. Our data indicate that TRX*o*1 regulates the expression and activity of target proteins such as ATG4 protease and the expression and protein level of ATG8, both of which are involved in the autophagy process. In fact, in vitro assays demonstrate the redox regulation of ATG4 and its interaction with the TRX*o*1 protein, suggesting that similar redox regulation of ATG4 by TRX may occur in vivo in the overexpressing TRX*o*1 cells.

## 2. Materials and Methods

### 2.1. Transformation of Tobacco BY-2 Cells

Stable PsTRX*o*1 over-expressing (OEX) lines of *Nicotiana tabacum* ‘Bright Yellow’ (TBY-2) suspension cells were generated as previously reported [28]. Two of these independent OEX lines (OEX1 and OEX2, containing GFP as a transformation marker) and a control line (without TRX*o*1 overexpression but containing GFP as a transformation marker) were used. The suspension of tobacco cells was routinely propagated and cultured at 26 °C and a stationary culture of 7 days of growth was diluted 4:100 (*v*/*v*) in fresh medium.

### 2.2. Cell Viability

Cell viability was calculated as the percentage of cells that did not stain with trypan blue [28]. An aliquot of cell suspension was transferred to a test tube containing 50 % (*v*/*v*) trypan blue solution. After 5–10 min, the trypan blue cell suspension mixture was transferred to a microscope slide and viable (unstained) and non-viable (blue-stained) cells were counted. For each sample, 5000 cells were scored, and four independent experiments were performed. Cells were visualized using a Leica DM6 microscope equipped with a digital MC 190 HD camera. 

### 2.3. H_2_O_2_ Treatment

Cells from the three lines at day 5 of growth in the exponential phase were exposed to 35 mM H_2_O_2_ (final concentration) as previously described [28]. At the indicated time points, cell aliquots were collected for analysis either by filtration on Whatman 3M paper or by centrifugation at 10,000× *g* for 10 min at 25 °C.

### 2.4. Incubation in the Presence of Autophagy Inhibitors

Two milliliters of tobacco culture at 5 days of growth were placed in ELISA plates and H_2_O_2_ was added at a final concentration of 35 mM in the absence or presence of either 10 μM E-64d (Aloxistatin, a cysteine protease inhibitor, Sigma, Merck, Darmstadt, Germany) (dissolved in methanol) or 10 μM E-64d plus 5 mM 3-MA (3-methyl adenine, an inhibitor of class III phosphatidylinositol 3-kinase PI3K, Sigma, Merck, Darmstadt, Germany) (dissolved in MilliQ water), as described in Takasutka et al. [32]. As a control for E-64d treatment, 1% methanol (*v*/*v*) was added to the cells. The cells were further cultured on a rotary shaker at 118 rpm at 26 °C in darkness and cell viability and autophagy analysis were carried out at the indicated times. 

### 2.5. Protein Extracts

Suspended cells were obtained with vacuum Kitasato flasks, weighed, and immediately frozen with liquid N_2_ and stored at −80 °C until use. The samples (3 g) were homogenized with a pre-cooled mortar and pestle in 3 mL of extraction buffer containing 50 mM HEPES-KOH, 5 mM DTT (1,4-dithiothreitol), 0.5 mM PMSF (phenylmethanesulfonyl fluoride), 1 mM EDTA (ethylenedinitrilotetraacetic acid), and 0.1% Triton X-100 (*v*/*v*), pH 7.4. The mixture was sonicated with 30 pulses in a SONICLAB at 70% amplitude and finally centrifuged at 16,000× *g* at 4 °C for 15 min. The supernatant was obtained, and the protein content was estimated by the Bradford [33] method.

### 2.6. Quantitative Real-Time PCR

The cells were sedimented by centrifugation and, after removing the residual medium, the fresh weight was determined, and the cells were frozen in liquid N_2_ and stored at −80 °C until analysis. RNA was isolated from cell sediment using the RNeasy Plant Mini Kit (Qiagen, Hilde, Germany) following the manufacturer’s protocol. cDNA was obtained from RNA by using the QuantiTect^®^Reverse Transcription Kit (Qiagen, Hilde, Germany) according to the manufacturer’s instructions. RNA and cDNA were quantified in an ND-1000 spectrophotometer (NanoDrop, Wilmington, NC, USA). qPCR was performed on a QuantStudio 5 Real Time PCR System (Applied Biosystems by Thermo Fisher Scientific, Bleiswijk, The Netherlands) using Power SYBR Green V PCR Master Mix (Applied Biosystems by Thermo Fisher, Madrid Spain) in the CAID Service of the University of Murcia. Each reaction was performed in triplicate using the following conditions: 2 min at 50 °C, 10 min activation at 95 °C, and 40 cycles of amplification (15 s at 95 °C; 1 min at 60 °C), followed by the melting curve. The relative quantity was calculated using the comparative Ct method [34]. The specific primers were designed using Primer Express v 2.0 (Thermo Fisher, Madrid, Spain) using the sequence from the data bank *NtATG4 (*accession number KR336561), listed in Supplementary Table S1. The expression of the *Actin 8* (*ACT8* GQ339768) gene was used to normalize the data. 

### 2.7. SDS–PAGE and Western Blot of Autophagy Markers

For Western blot analysis, SDS–PAGE was carried out on AnykD (BioRad, Madrid, Spain) polyacrylamide gels, and 40 μg of protein extracts were transferred to nitrocellulose membranes using a semi-dry Trans-Blot cell (BioRad, Madrid, Spain). Membranes were used for cross-recognition assays using polyclonal antibodies anti-CrATG4 (Agrisera, Vännäs, Sweden) or anti-CrATG8A (Agrisera, Vännäs, Sweden). For immunodetection in membranes, a goat anti-rabbit IgG–horseradish peroxidase conjugate (Santa Cruz Biotechnology, Heidelberg, Germany) was used as the secondary antibody, together with an enhanced chemiluminescence kit (Supersignal^TM^ West Picoplus, Fisher Scientific, Madrid, Spain). Densitometry of the different bands was performed using an image analyzer (Amersham Imager 600, GE Healthcare, Barcelona, Spain) and the Amersham ImageQuanTL 8.1 Program (Cytiva, Barcelona, Spain). Ponceau staining was used to correct the loading control in each line.

### 2.8. ATG4 Cleavage Assay

ATG4 activity was measured by electrophoretic separation of the cleaved ATG8 substrate (processed pATG8) containing a C-terminal tag after the cleavage site. For this, a recombinant human GABARAP protein (Fc Chimera, ATG8-Fc, Abcam, Cambridge, UK) also containing an N-terminal 6His-tag was used, as previously described [35]. Cleaved ATG8 was detected by Western blot using an anti-His antibody as described below.

To analyze the effect of reducing and oxidizing conditions on the in vitro HsATG4 activity, a typical reaction mixture contained 0.22 µg of recombinant HsATG4B treated under reducing (15 µM DTT for 5 min at 25 °C) or oxidizing (1 mM H_2_O_2_ or 3 mM H_2_O_2_ for 5 min at 25 °C) conditions in TBSK buffer (50 mM Tris-HCl, 140 mM NaCl, 30 mM KCl, pH 8.0) and, where indicated, under reducing conditions followed by oxidizing ones. The reaction mixtures were then incubated with 0.48 µg ATG8-Fc for 45 min at 30 °C and stopped by the addition of reductant-free Laemmli sample buffer followed by incubation for 5 min at 95 °C. Proteins were resolved on TGX 4–20% SDS-PAGE gels (BioRad, Madrid, Spain). Two negative controls were performed using only HsATG4B in the absence of ATG8-Fc, and with ATG8-Fc in the absence of HsATG4B. Western blot analysis using the primary antibody monoclonal anti-6His (Agrisera, Vännäs, Sweden, 1:5000) and anti-mouse IgG HRP secondary antibody (Abcam, Cambridge, UK 1: 5000) followed by immunodetection using a chemiluminescence kit (Supersignal^TM^ West Picoplus, Fisher Scientific, Madrid, Spain) according to the manufacturer’s instructions allowed the detection of the reaction product of the ATG4 activity as the processed ATG8 (pATG8).

To analyze the HsATG4 in vitro activity in the presence of the thioredoxin system, HsATG4B previously reduced by 15 µM DTT was treated with 1 mM H_2_O_2_ as described above. This oxidized HsATG4B was then reduced using 2.5 µg PsTRX*o*1 (or a mutated version mutPsTRX*o*1C37S [23], 1.5 µM AtNTRA (NADPH-dependent thioredoxin reductase A), and 0.04 mM NADPH in TBSK buffer [26]. Several control reactions were performed in the absence of any of these components or using 10 µM DTT instead of the NADPH/TRX*o*1/NTRA system. The different reaction mixtures were then incubated with 0.48 µg ATG8-Fc for 45 min at 30 ºC before adding Laemmli sample buffer and continuing SDS-PAGE as described above. The cleaved product of ATG8-Fc was identified after Western blot as described. 

For the in vivo ATG4 activity, we followed a previously described method [35], whereby 50 μg of TBY-2 cellular protein extracts were incubated with recombinant ATG8-Fc (0.6 μg) in TBSK reaction buffer and 100 μM DTT at 30 °C for 45 min. The reaction wasstopped by adding sample buffer (reductant-free Laemmli buffer) and 5 min of boiling, after which the samples were resolved on AnykD (BioRad, Madrid, Spain) gels by SDS–PAGE and subsequently analyzed by Western blot using the anti-6His antibody as described above. A negative control was performed on a cellular extract (OEX1 line) without ATG8-Fc substrate to reveal non-specific signals.

### 2.9. Visualization of Autolysosomes

TBY-2 suspension tobacco cells (200 µL) were centrifuged at 4000× *g* for 5 min and the pellet was resuspended in 200 µL of 100 mM potassium phosphate buffer (PK) pH 6.5 containing 1 µM of Lysotracker Deep Red (Invitrogen, Thermo Fisher, Madrid, Spain) as previously described [36]. The mixture was incubated in the dark and at room temperature for 6 min. Subsequently, the preparation was centrifuged at 1000× *g* for 1 min, the supernatant was discarded, and the pellet was washed three times with PK buffer, pH 6.5 at 1000× *g* for one minute, and the final precipitate was resuspended in 150 µL of the same buffer. Fifty microliters were added to the slide and the structures were visualized in a Leica DM6 motorized fluorescence microscope equipped with Nomarski optic and a digital Hamamatsu C11440 camera. For the detection of autolysosomes, the preparations were observed using a band pass (BP) 545–580, DM 600, and a BA filter of 610. At least ten fields (approximately 20 cells per field) were evaluated in each of the three independent experiments carried out. Image analysis was performed using ImageJ software (Softonic, NIH, Bethesda, MD, USA).

### 2.10. Immunolocalization of ATG8

Fluorescence microscopy was used for the immunolocalization of ATG8. TBY-2 cells were fixed and stained as described by Voitsekhovskaja et al. [36] with some modifications. Cells were fixed with 1% glutaraldehyde in MS medium and treated with a mixture of cellulose (5 U/mL, Onozuka, Duchefa Biochemie, Haarlem, The Netherlands ), 10% hemicellulose (Macerozyme R-10, Duchefa Biochemie, Haarlem, The Netherlands), and 5 U/mL pectinase (Sigma, Merck, Darmstadt, Germany) in osmotic media. After washing and treatment with 0.3% Triton X-100 and blocking with 5% BSA, cells were incubated with the primary antibody anti-ATG8 (1:1000, Agrisera, Vännäs, Sweden) for 1 h and then with the Alexa-568-conjugated secondary antibody (1:100, Molecular Probes, Thermo Fisher, Madrid, Spain) for 1 h before washing four times. Cells were observed with a Leica DM6 motorized fluorescence microscope equipped with a digital camera with an excitation wavelength of 579 nm and emission of 603 nm, using a rhodamine (RHO) filter and a 100X/1.30 oil objective. At least ten fields (approximately 20 cells per field) were evaluated in each of the three independent experiments carried out. When indicated, images were treated with Huygens Essential Microscopy Analysis software (SVI, Stockton, CA, USA) at the University of Murcia (Scientific Image Treatment’s Service) for better visualization of the vesicles. Image analysis was performed using ImageJ software (Softonic, NIH, Bethesda, MD, USA).

### 2.11. Protein Dot-Blot

Recombinant proteins PsTRX*o*1 (1.5 µg, [24], HsATG4B (Abcam, 2.3 µg), and bovine serum albumin (BSA) (1.5 µg, Sigma, Merck, Darmstadt, Germany) as negative control were spotted on 6 pieces of the membrane (2 membranes for each protein). The membranes were incubated in TBS for 5 min and then blocked in TBST buffer (25 mM Tris-HCl pH 7.5 containing 1% (*w*/*v*) BSA and 0.1% (*v*/*v*) Tween-20 (Sigma, Merck, Darmstadt, Germany) for one hour at room temperature. Then, the membranes were overlaid with 0.2 µg of recombinant PsTRX*o*1 (membranes A) or 0.1 µg of recombinant HsATG4B (membranes B) in TBST and incubated for 2 h at room temperature. The A membranes were incubated with the polyclonal anti-PsTRX*o*1 (1:1000) [24] and B membranes with monoclonal anti-CrATG4B (Agrisera, Vännäs, Sweden, 1:5000) diluted *v*/*v* in TBST, overnight at 4 °C with stirring. Subsequently, the membranes were washed five times with TBST for 5 min at room temperature and incubated with rabbit Santa Cruz anti-HRP 1:30,000 (*v*/*v* in TBST) for TRX*o*1 and anti-HRP mouse 1:2000 (*v*/*v* in TBST) for ATG4B. Finally, the membranes were washed five times with TBST for 5 min at room temperature and once again with TBS. Immunodetection was performed using a chemiluminescence kit (Supersignal^TM^ West Picoplus, Fisher Scientific, Madrid, Spain) according to the manufacturer’s instructions. 

### 2.12. Co-Immunoprecipitation PsTRXo1/His-HsATG4

Co-immunoprecipitation of the PsTRX*o*1 and HisHsATG4 proteins was performed using the commercial µMACS Anti-His MicroBeads kit (MACS Miltenyi Biotec, Madrid, Spain). To this end, recombinant proteins capable of interacting (4 µg His-HsATG4B and 8 µg PsTRX*o*1) were diluted in 500 µL of “lysis buffer” provided by the kit. A 50 µL fraction was kept for use as a loading control containing both proteins (Input in the gel). Fifty microliters of Anti-His magnetic µbeads were added and the protein mixture was incubated for 2 h at 4 °C under mild stirring.

The columns provided by the kit were placed according to the manufacturer’s instructions on the designed magnetic support and they were prepared by passing 200 µL of “lysis buffer” from the kit. Once the incubation time between the proteins and the Anti-His µbeads was over, the solution was deposited into the columns and washed 4 times with 200 µL of “wash buffer 1” and then rinsed with 100 µL of “wash buffer 2” (fraction named as Wash in the gel). Then, 20 µL of “elution buffer” preheated at 95 °C was added to the column and incubated for 5 min at room temperature Fifty microliters of preheated elution buffer at 95 °C was added to the column and the eluate was considered as co-immunoprecipitated (CoIP) PsTRX*o*1/His-HsATG4B proteins. Samples were analyzed by Western blot after SDS-PAGE using anti-His. Then the membrane was incubated in TBST at room temperature for 1 h and revealed for the presence of PsTRX*o*1 using anti-PsTRX*o*1.

### 2.13. Statistical Analysis

Each experiment was repeated at least three times with three biological replicates per treatment for each genotype. The figures show data from a typical experiment if not otherwise indicated. Values presented are mean ± standard error. Different letters indicate significant differences (*p* < 0.05) among genotypes in each condition according to Tukey’s-b test using SPSS software (IBMVR SPSSVR Statistics 19, New York, USA). The asterisks indicate significant differences of each genotype after the H_2_O_2_ treatment compared to the control condition using Student’s *t*-test at *p* < 0.05 (*), *p* < 0.005 (**), or *p* < 0.001 (***).

## 3. Results

### 3.1. Overexpressing PsTRXo1 Cells Present High Viability and Elevated Levels of Autophagy Markers

**Figure 1:**
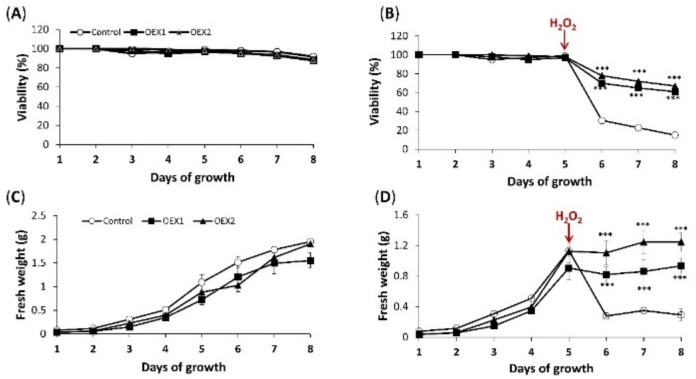
Cell viability and fresh weight of control and two lines of overexpressing PsTRX*o*1 (OEX1 and OEX2) TBY-2 lines (**A**,**C**) and after 35 mM H_2_O_2_ treatment (**B**,**D**). Cell viability (percentage) was measured using Trypan Blue dye during the 8 days of culture. H_2_O_2_ was added at day 5 of growth as indicated by the arrows. Values represent mean ± standard error of four independent experiments. Differences of OEX cells values related to the control line in each time point in (**A**,**C**) were not significant according to Student’s *t*-test (*p* < 0.05, n > 1000 cells) while *** represent significant differences in (**B**,**D**) (*p* < 0.001, n > 1000 cells).

Overexpression of PsTRX*o*1 in tobacco BY-2 cells increases viability after oxidative treatment with 35 mM H_2_O_2,_ as previously reported by our group, due to increased antioxidant status and delayed cell death [28]. In the present work, we analyze whether autophagy might also be collaborating with those processes to increase cell viability. First of all, we confirmed that similar to that found in our previous work [28], a control line and two overexpressing *Pstrxo1* lines (OEX1 and OEX2) presented similar viability (close to 100%) during culture growth up to 8 days (Figure 1A). We also found that, as before, a 35 mM H_2_O_2_ treatment at day 5 of growth provoked greater mortality in the non-overexpressing cells (control line)—around 30% viability after 24 h (Figure 1B) compared to the 70% observed in the two OEX lines. The fresh weight was similar in the three lines during growth in control conditions (Figure 1C), and this parameter decreased sharply in non-overexpressing cells while OEX lines maintained a high fresh weight after the H_2_O_2_ treatment (Figure 1D).

The difference observed in viability 24 h after oxidative treatment among cell lines led us to analyze how different autophagy markers were affected by the overexpression of PsTRX*o*1 and by the stress applied (day 6 of growth). 

**Figure 2:**
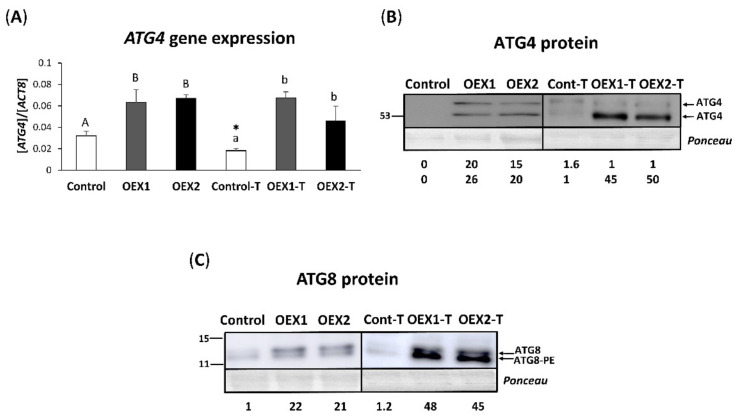
*ATG4* (**A**) gene expression and ATG4 (**B**) and ATG8 (**C**) protein content in control and two overexpressing *Pstrxo1* (OEX) TBY-2 lines at day 6 of growth (left panel) and 24 h after 35 mM H_2_O_2_ treatment (treated cells (-T), right panel). (**A**) mRNA level was determined by real-time qPCR. Values are expressed as relative expression against the *NtActin8 (ACT8)* gene. Bars show means ± standard error of three independent experiments with three biological replicates in each one and three technical replicates. Different letters indicate significant differences (*p* < 0.05) between genotypes in each condition according to Tukey’s test, and the asterisks indicate significant differences for each genotype after the H_2_O_2_ treatment compared with the non-treated cells, as determined by Student’s *t*-test (*p* < 0.05). (**B**,**C**) Representative Western blot of ATG4 and ATG8 protein of cellular extracts separated by SDS–PAGE and visualized by chemiluminescence, pointing to the non-lipidated and lipidated (PE) forms of the protein. Pounceau staining of the membrane was used to correct the loading. Values under figures are the mean of three experiments quantified after densitometry (numbers below show the sum of both forms for ATG8 while each band for ATG4) and refer to the lowest band intensity whose value is 1.

The analysis of *ATG4* gene expression under control conditions (Figure 2A) revealed that the transformation with PsTRX*o*1 increased the expression of *ATG4*. The H_2_O_2_ treatment affected only the non-overexpressing cells (Cont-T), decreasing *ATG4* gene expression, showing that the overexpression of PsTRX*o*1 allows the maintenance of high levels of *ATG4* in OEX cells. 

Western blot analysis of the ATG4 protein revealed the presence of two bands recognized by the antibody at around the described molecular weight of the isoforms X1 and X2 of the tobacco proteins, 53 and 57 kDa (Uniprot database: XP_016513796 and XP_016513797) (Figure 2B). Western blot analysis of the ATG8 content in non-overexpressing cells showed two bands of different molecular weights (Figure 2C) corresponding to the free cytosolic forms, namely ATG8 around 13 kDa and below it the lipidated form ATG8-PE (PE: Phosphatidylethanolamine) as previously described [37]. Overexpression of PsTRX*o*1 increased the level of both proteins, ATG8 (non-lipidated and lipidated forms) and ATG4 (left panels). In fact, ATG4 was detected only in OEX cells and not in the control line under non-oxidative conditions. The H_2_O_2_ treatment increased ATG8 content particularly in the OEX lines (more than double) and especially the lipidated ATG8-PE form (Figure 2C). The oxidative treatment induced the accumulation of both ATG4 isoforms in the Control (Cont-T) line but more intensively in the OEX-T lines, which presented a higher protein content at 53 kDa (Figure 2B). These results show that both the overexpression of PsTRX*o*1 and H_2_O_2_ treatment provoked an increase in ATG8 and ATG4 autophagy markers, with OEX-treated cells being the ones presenting higher levels than non-overexpressing cells. 

**Figure 3:**
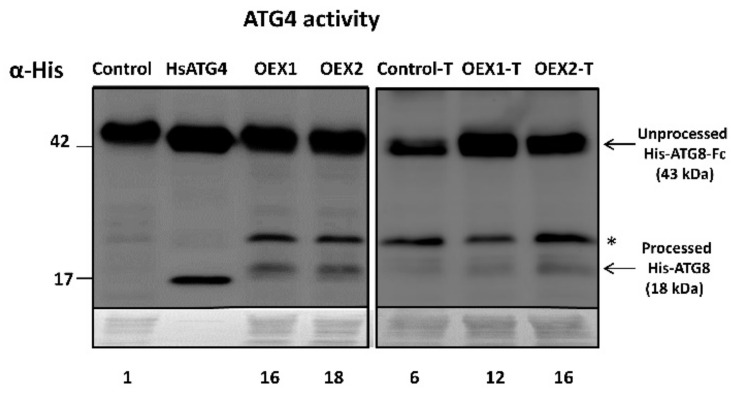
Endogenous ATG4 processing activity of control and two overexpressing *Pstrxo1* (OEX) TBY-2 lines at day 6 of growth (**left panel**) and 24 h after 35 mM H_2_O_2_ treatment (treated cells (-T), **right panel**). ATG4 activity of cellular extracts in the presence of DTT, monitored by following the cleavage of recombinant quimeric His tag ATG8 substrate from the unprocessed (His.ATG8-Fc) to the processed His-ATG8) forms (indicated by arrowheads) by Western blot analysis using anti-His antibody. The activity of recombinant HsATG4 is shown as positive control. Asterisk indicates a non-specific band. Values are the mean of three experiments quantified after densitometry of the detected 18 kDa bands (see numbers below the membrane relatives to the signal in the control line).

To check whether changes in expression and protein levels correlated with activity levels, endogenous ATG4 activity was assayed in the three cell lines in the presence of DTT. In the absence of the H_2_O_2_ treatment and parallel to the protein content, the control line presented low ATG4 activity while overexpressing *Pstrxo1* cells showed high activity, as seen from the presence of a cleavage product at around 18 kDa (Figure 3, left pannel). The activity of recombinant HsATG4 was measured as a positive control. After the H_2_O_2_ treatment, similar to that found for protein contents, OEX lines maintained higher ATG4 activity, being around double than that of the control line, which showed a 6-fold increase. A non-specific band appeared around 24 kDa with higher intensity in the overexpressing lines (asterisk in Figure 3) as resulting from the incubation of cells without the ATG8-Fc substrate (Figure S1).

### 3.2. Autophagy Is Related to Cell Viability

After demonstrating that the overexpression of PsTRX*o*1 induced an increase in cell viability after H_2_O_2_ treatment with the cells presenting higher autophagy markers, such as ATG4 and ATG8, than non-overexpressing ones, we wondered whether this increased viability and higher levels of autophagy markers were accompanied by activation of the autophagy process. In this way, autophagy could act as a protective mechanism probably eliminating damaged components in response to the oxidative stress situation, thus increasing viability. In order to analyze whether autophagy was involved in the higher viability of the OEX lines under oxidative conditions, in addition to the treatments used in Figure 1, we measured viability in the presence of two autophagy inhibitors. E-64d, a Cys-protease inhibitor that functions by suppressing lysosomal proteases and results in the accumulation of autophagosome-and (auto)lysosome-like structures and 3-methyladenine (3-MA), which reverts the E-64d effect in tobacco cells subjected to induced autophagy, were investigated [5,32].

**Figure 4:**
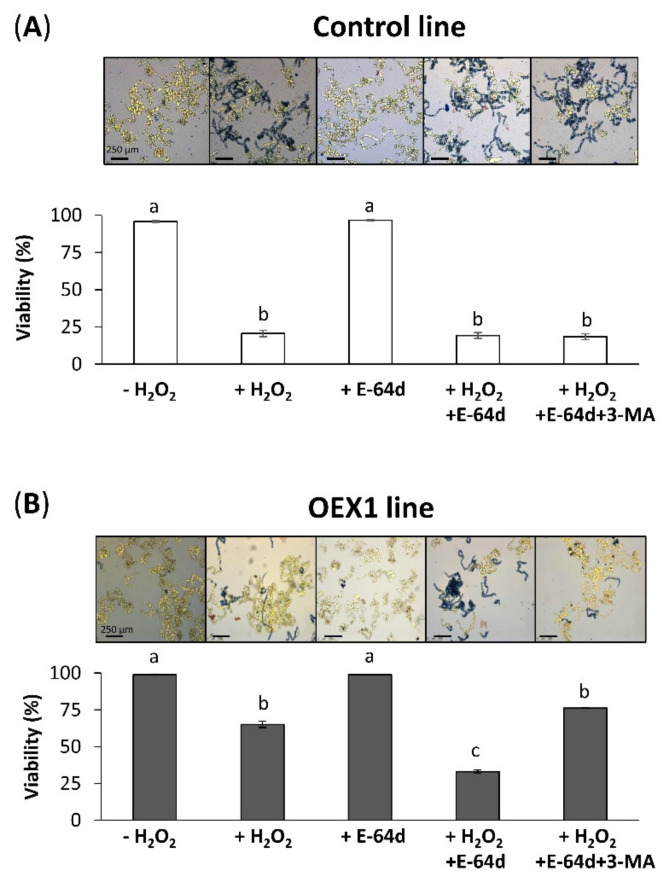
Cell viability of the control (**A**) and *Pstrxo1* transformed (**B**) TBY-2 lines 24 h after 35 mM H_2_O_2_ treatment in the absence and presence of the autophagy inhibitors E-64d and 3-MA. Cell viability (percentage) without treatment (-) and after treatment (+) with H_2_O_2_, E-64d, H_2_O_2_ + E-64d, and H_2_O_2_ + E-64d + 3-MA was tested using Trypan Blue dye 24 h after the respective treatments. Values represent mean ± standard error of four independent experiments. Different letters indicate significant differences (*p* < 0.05) among treatments according to Tukey’s test.

Similar to Figure 1, we first measured the viability of the control cells in the absence of, or 24 h after, H_2_O_2_ oxidative treatment (+H_2_O_2_). The H_2_O_2_ treatment provoked a decrease in viability from 96% to 20% in the control line (Figure 4A) and E-64d (+E-64d) alone did not affect the viability. When both H_2_O_2_ and E-64d alone and in combination with 3-MA were applied to the cell culture, no effect was observed related to the + H_2_O_2_ treatment. These results indicate that autophagy is not involved in the viability of control cells under oxidative conditions. A different effect was observed in OEX1 cells, which showed a decrease in viability from 99% to 65% with the oxidative treatment (Figure 4B). Again, the inhibitor E-64d alone did not affect their viability. Interestingly, when H_2_O_2_ plus E-64d were applied to the OEX line, a marked decrease in cell viability was observed (around 33%), which was reverted in the presence of 3-MA (last picture in Figure 4B), again leading to high cell viability (76%), indicating an autophagy-dependent cell survival situation. We then investigated whether the effect of the two inhibitors was observed in the OEX1 line 48 h after H_2_O_2_ treatment, and similar results were obtained (Figure S2), so the effect of inhibitors lasted for at least this time. Similar results were obtained with the OEX2 line (data not shown). We did not measure the effect of inhibitors in the control line 48 h after oxidative treatment because they were not alive at this time. We also determined that 3-MA did not affect OEX1 line viability when applied in the absence of E-64d (Figure S3) in both the absence or presence of H_2_O_2_ 24 h after oxidative treatment, while it decreased viability around 5% 48 h after.

All these experiments point to a relationship between autophagy and viability in the cell response to the oxidative situation occurring 24 h/48 h after H_2_O_2_ treatment, only when PsTRX*o*1 is over-expressed.

### 3.3. Overexpressing Pstrxo1 Cells Present Lytic Vesicles 24 h and 48 h after Oxidative Treatment

To further our knowledge of the autophagy process taking place in the overexpressing cells under the oxidative H_2_O_2_ treatment, we decided to observe the presence of autophagic structures. Autophagy can be analyzed using the acidotropic fluorescent dye Lysotracker Deep Red (LDR), which labels autolysosomes. E-64d is an inhibitor that blocks the transfer of autolysosomes to the vacuole, allowing their accumulation, blocking the autophagy flux, and enabling the structures to be stained by LDR and visualized. 

**Figure 5:**
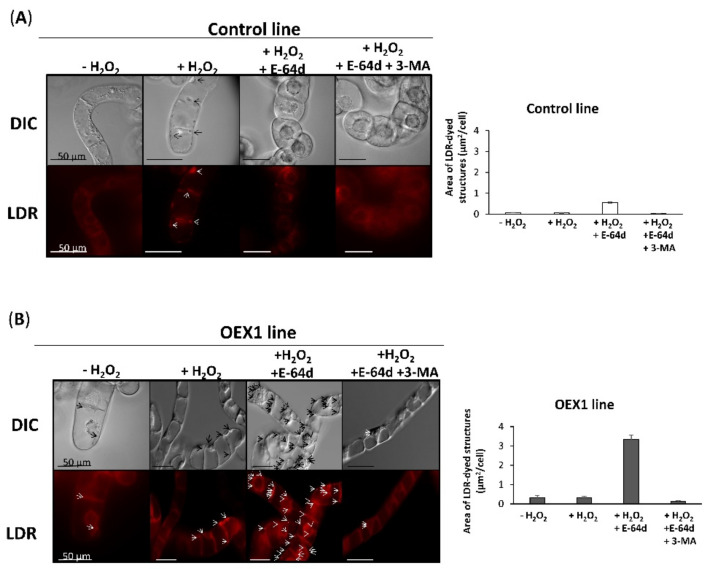
Visualization of H_2_O_2_-induced formation of autolysosomes in control (**A**) and overexpressing *Pstrxo1* (OEX1) (**B**) tobacco BY-2 cells. Differential interference contrast (DIC) images and fluorescence images of LDR-dyed structures are representative of three independent experiments. Five-day-old TBY-2 cells were treated with 35 mM H_2_O_2_ alone or in combination with E-64d or E-64d plus 3-MA and observed after 24 h under a fluorescence microscope equipped with a Nomarski optic. Arrows indicate some signals of autolysosomes (red). Scale bars: 50 μm.

The analysis of the presence of vesicles was carried out in control and OEX1 lines (Figure 5A,B, respectively) 24 h after the H_2_O_2_ treatment in the presence of the inhibitor E-64d using a fluorescence microscope with a Nomarski optic (DIC). An accumulation of vesicles visualized in both DIC- and LDR-labelled cells was observed mainly in OEX1 cells when compared to control cells after the H_2_O_2_ + E-64d treatment (third column in Figure 5). It seems that some autophagosomes are constitutively accumulated in OEX lines due to the detection of LDR signals in cells cultured in the absence or presence of H_2_O_2_ without the inhibitor (the first and second columns in Figure 5) or when the compound 3-MA was also present (last column in Figure 5). This result indicated that the E-64d inhibitor principally affected OEX1 cells blocking the autophagy process and inducing the accumulation of vesicles. The accumulation was observed even 48 h after the treatments in OEX1 cells (Figure S4). All these results demonstrated the occurrence of an autophagy flux evident by the accumulation of autolysosomes in the transformed PsTRX*o*1 cells subjected to the H_2_O_2_ treatment. 

### 3.4. Overexpressing Pstrxo1 Cells Accumulate ATG8 Protein Detected by Immunofluorescence 24 h after Oxidative Treatment

**Figure 6:**
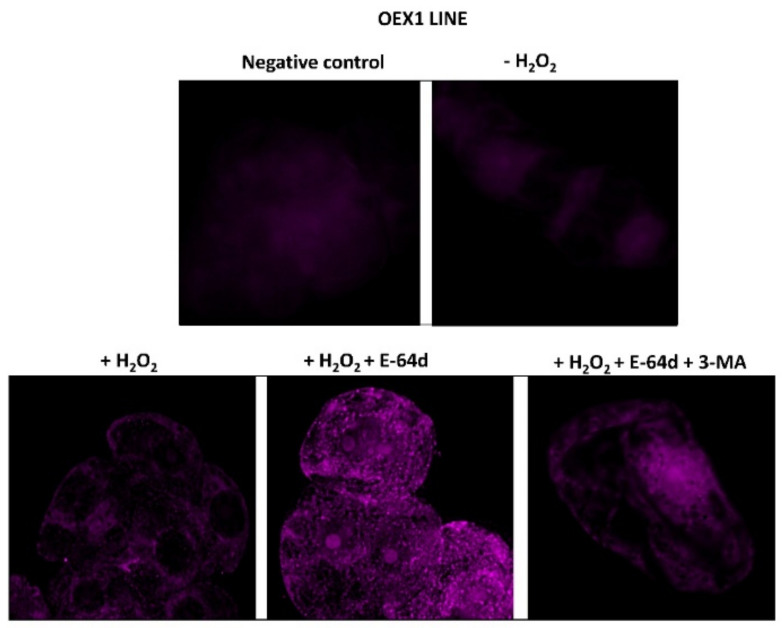
Immunodetection of ATG8 in overexpressing *Pstrxo1* OEX1 tobacco BY-2 cells treated with 35 mM H_2_O_2_. Five-day-old overexpressing (OEX1) *Pstrxo1* TBY-2 cells were treated in the absence (-) and presence (+) 35 mM H_2_O_2_ alone or in combination with E-64d and 3-MA and collected and processed after 24 h for immunofluorescence microscopy, as described in Material and Methods. Fluorescence images of the signal recognized by the anti-CrATG8 antibody (purple punctates) are shown as representative of three independent experiments after using the Huygens Essential Microscopy Analysis software. A negative control without incubation with the primary antibody is also presented.

In order to corroborate the existence of an autophagy flux, the antiCrATG8 antibody was used to examine the presence of this protein by immunofluorescence (IF) in the OEX1 line treated with H_2_O_2_ in the absence and presence of E-64d and in the presence of E-64d plus 3-MA. A high IF signal was detected in cells treated with H_2_O_2_ plus E-64d compared with that seen in cells treated with the oxidant in the absence of the inhibitor (Figure 6), corroborating the existence of autophagosomes containing the ATG8 protein in these H_2_O_2_-treated OEX1 cells. This ATG8 accumulation was reverted by using 3-MA in combination with E-64d. Similar results were obtained in the OEX2 line (data not shown). 

**Figure 7:**
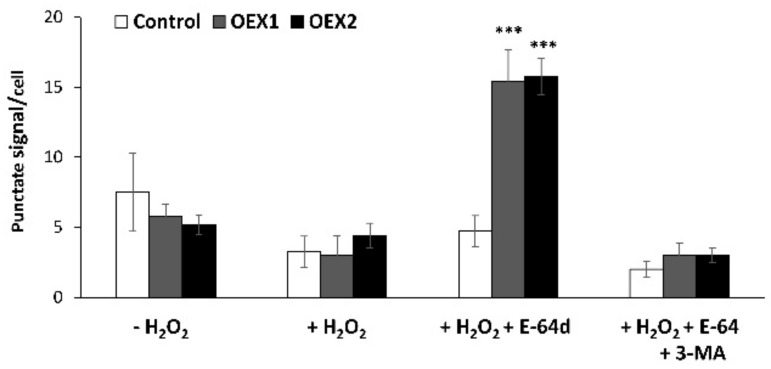
Quantification of ATG8 after immunofluorescence analysis. Number of immunofluorescent punctate signals per cell after immunodetection of ATG8 in Control and two overexpressing *Pstrxo1* (OEX) tobacco BY-2 cells 24 h after treatment with 35 mM H_2_O_2_ and in the presence of E-64d or E-64d plus 3-MA as described in Material and Methods. The asterisks *** indicate significant differences of each genotype compared to the H_2_O_2_-treated cells (+H_2_O_2_), as seen from Student’s *t*-test (*p* < 0.001).

The punctate signals per cell were quantified in control and two OEX lines as the measurement of the autophagy flux, and the values are presented in Figure 7. After analyzing 20 cells per genotype and treatment, only the OEX lines presented a high accumulation of ATG8 24 after the H_2_O_2_ + E-64d treatment, with the values being three times higher in these lines than in the H_2_O_2_-treated cells. 

### 3.5. Autophagy Is Evident in OEX Cells and Not in Control Cells 14 h after Oxidative Treatment

**Figure 8:**
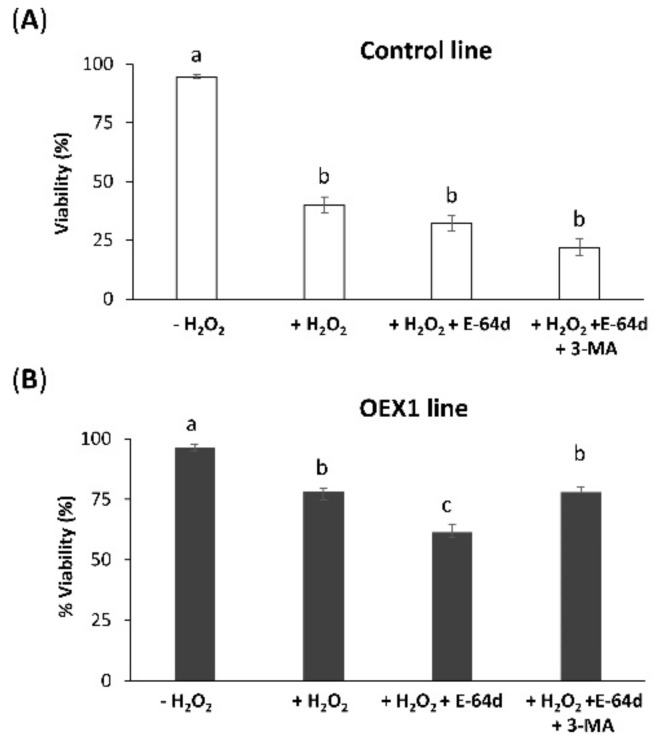
Cell viability of the control (**A**) and overexpressing *Pstrxo1* TBY-2 OEX1 (**B**) lines 14 h after treatment with 35 mM H_2_O_2_ in the absence and presence of the autophagy inhibitors E-64d and 3-MA. Cell viability (percentage) of cells without treatment (-) and after treatment with H_2_O_2_, H_2_O_2_ + E-64d, and H_2_O_2_ + E-64d + 3-MA was tested using Trypan Blue dye 14 h after oxidative treatment. Values represent mean ± standard error of four independent experiments. Different letters indicate significant differences (*p* < 0.05) among treatments according to Tukey’s test.

Based on the observed low viability of the control non-overexpressing line 24 h after H_2_O_2_ treatment and in order to assess the possible occurrence of an increase in autophagy as a short-term response in this control line, we attempted to ascertain the point these cells presented higher viability than at 24 h, choosing 14 h post H_2_O_2_ treatment. At this time, the control line presented 40% viability and the OEX lines around 80% (Figure 8A). Using E-64d, no significant effect on cell viability was observed in the control line while in the OEX lines, the viability decreased significantly to 60% (Figure 8B). 3-MA did not affect viability in the control line after the oxidative treatment while it reverted the effect of E-64d in the OEX1 line. Similar results were obtained in the OEX2 line (data not shown). We also tested the effect of the inhibitors at 2 h after the H_2_O_2_ treatment finding no effect on cell viability in any of the three lines (Figure S5).

**Figure 9:**
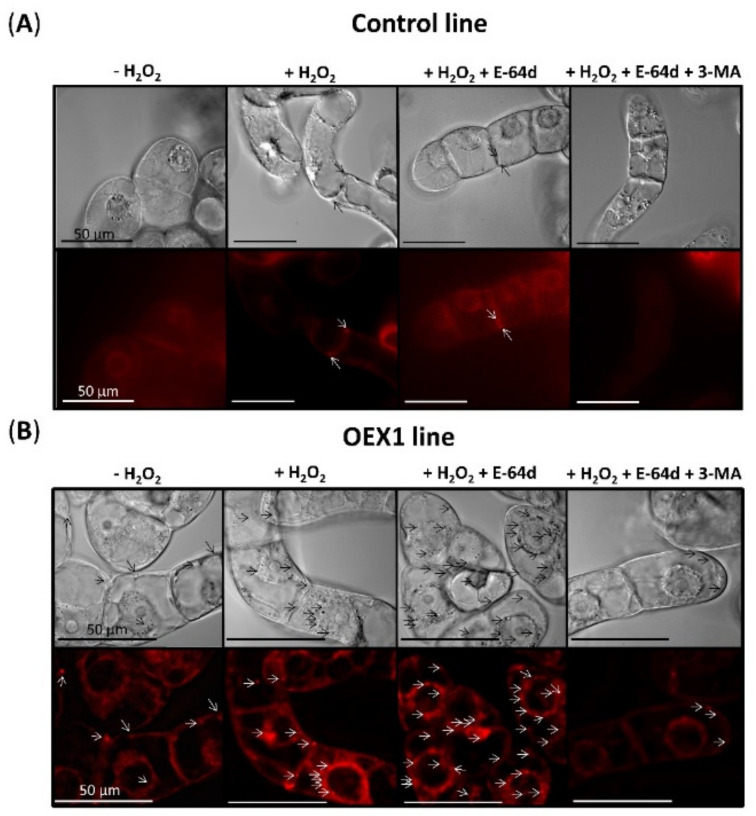
Visualization of H_2_O_2_-induced autolysosomes formation in control (**A**) and overexpressing *Pstrxo1* (OEX1) (**B**) tobacco BY-2 cells. Differential interference contrast (DIC) images and fluorescence images of Lysotracker Deep-Red-stained structures are shown as representative of three independent experiments. Five-day-old TBY-2 cells were treated with 35 mM H_2_O_2_ alone or in combination with E-64d or E-64d and 3-MA and observed after 14 h under a fluorescence microscope equipped with a Nomarski optic. Arrow heads indicate some punctate signals of autolysosomes (red). Scale bars: 50 μm.

These results suggest the lack of autophagy in the control non-overexpressing *Pstrxo1* line in response to the oxidative condition at 14 h. To corroborate this, we analyzed the presence of autolysosomes 14 h after H_2_O_2_ treatment, in the absence and presence of the E-64d inhibitor. Similar to that observed for the effect of E-64d on cell viability, low-intensity fluorescence was accompanied by no autolysosome accumulation in the control line (Figure 9A) while higher intensity was observed, and these structures mainly accumulated in E-64d-treated cells of the OEX line 14 h after H_2_O_2_ treatment (Figure 9B). 

### 3.6. ATG4 and TRXo1 Interact In Vitro

One of the possible control mechanisms of the autophagy process may be the redox regulation by TRX of autophagy components such as certain ATG proteins, as previously reported for ATG4 in Chlamydomonas and yeast (described in the introduction section). To determine whether TRX could also regulate the ATG4 protein in plants, we first performed an experiment to analyze the possible interaction between both proteins using recombinant pea PsTRX*o*1 and human HsATG4B, and then studied any possible redox regulation in vitro of the ATG4 cleavage activity by the NTR/TRX*o*1 system.

**Figure 10:**
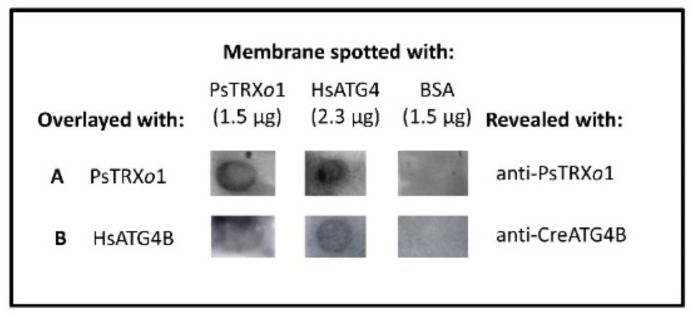
Representative protein gel dot blot analysis of the interaction between recombinant PsTRX*o*1 and HsATG4. Membranes were spotted with recombinant PsTRX*o*1, HsATG4B, and BSA (negative control) and were overlayed with TBST buffer containing the recombinant PsTRX*o*1 or HsATG4B proteins. The presence of the proteins was revealed using anti-PsTRX*o*1 or anti-CreATG4B antibodies, respectively.

To analyze the physical interaction of PsTRX*o*1 and HsATG4B, a protein dot blot experiment was carried out, spotting the proteins onto nitrocellulose membranes separately and also overlaying them separately. After extensive washing of the membrane, the presence of TRX*o*1 bound to spotted ATG4 was revealed by anti-PsTRX*o*1 (Figure 10 line A) and the presence of ATG4 bound to TRX*o*1 spotted on the filter was revealed by the anti-CreATG4 antibody (Figure 10 line B). As positive controls, the PsTRX*o*1 recombinant protein was spotted and revealed with anti-TRX*o*1 and HsATG4 was spotted and revealed with anti-ATG4 (Figure 10 lines A, first spot and B, second spot). The interaction appeared to be specific, since no signal was detected in positions corresponding to equivalent amounts of BSA, spotted as negative controls (membranes in the last column). These results confirm the in vitro physical interaction of PsTRX*o*1 and HsATG4B proteins.

**Figure 11:**
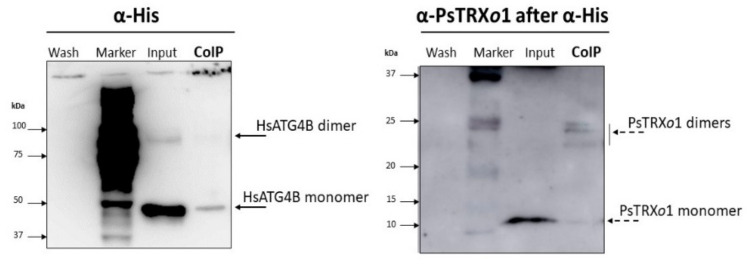
Western blot of the resulting Co-IP between His-HsATG4B and PsTRX*o*1 recombinant proteins revealed with anti-Histidine and anti-PsTRX*o*1 after mild stripping. The first lane contained the last wash before elution of the coimmunoprecipitated (CoIP) complexes from the microparticles to check the absence of any of the proteins. The second lane contained the Marker of molecular weights with the corresponding numbers in kDa. The input lane corresponds to a fraction of both proteins incubated before passing through the column and the last lane contained the CoIP proteins revealing the interaction between HsATG4B and PsTRX*o*1 in their monomeric and dimeric forms.

To corroborate the result obtained in the dot-blot trap assay, a Co-immunoprecipitation (CoIP) of recombinant His-HsATG4B and PsTRX*o*1 proteins was performed followed by Western blot analysis of the presence of both proteins using Anti-His MicroBeads. The last wash of the magnetic particles before elution of the column was loaded on the first lane of the gel (membrane on the left in Figure 11) to check the absence of the proteins not retained before elution (Wash fraction). The third lane contained a fraction of the solution in which the proteins were incubated before passing through the column (Input), which demonstrated the presence of monomeric and dimeric His-HsATG4B protein (as revealed by anti-His). The last lane contained the proteins eluted from the microparticles (CoIP), which mainly revealed the presence of the monomeric HsATG4B protein. The same membrane was then subjected to mild stripping and incubated with anti-PsTRX*o*1, revealing the presence of the monomeric (around 12.5 kDa) form of this protein in the input fraction and also in the last lane containing the CoIP eluted from the magnetic microparticles (membrane on the right in Figure 11) together with several dimeric forms around 24–25 kDa. The presence of both proteins in the eluted CoIP fraction confirmed the interaction between HsATG4B and PsTRX*o*1 proteins. Due to the low amount of TRXo1 in the eluted CoIP fraction, the membrane was revealed with anti-PsTRX*o*1 over a long duration for the detection of the protein, which also allowed the detection of the dimeric HsATG4B in the input fraction (see whole membranes in Supplemental Figure S6). 

### 3.7. ATG4 Activity Is Redox-Regulated

**Figure 12:**
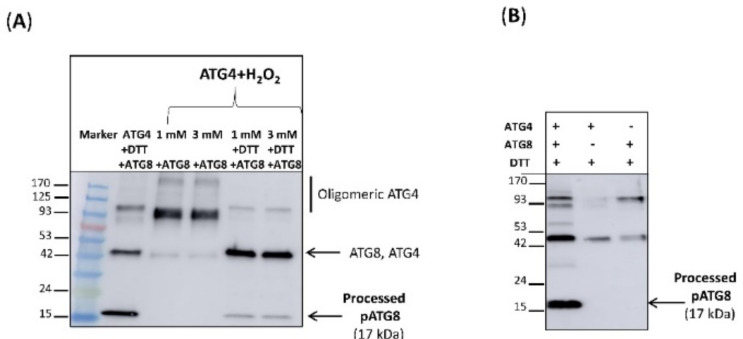
Regulation of ATG4 activity by oxidation and reduction. ATG4 activity was monitored by following the cleavage of recombinant quimeric His tag ATG8 substrate from the unprocessed to the processed pATG8 forms (indicated by arrowheads) by Western blot analysis using anti-His antibody. (**A**) His tag HsATG4B was incubated with DTT (lane 2), 1 mM (lane 3) and 3 mM (lane 4) of H_2_O_2_, and 1 mM or 3 mM H_2_O_2_ followed by DTT (lanes 4 and 5, respectively) prior to their incubation with the substrate quimeric ATG8. Marker (M) proteins were loaded in lane 1. (**B**) Control experiment for the activity of recombinant His-tag protein ATG4 in the presence of His-ATG8-Fc (lane 1) and negative controls with only His-ATG4 or His-ATG8-Fc proteins (lanes 2 and 3, respectively) after the DTT treatment (see Material and methods section for details).

After confirming the interaction between HsATG4 and PsTRX*o*1 in vitro, we tried to ascertain whether the HsATG4 protein might be redox regulated by PsTRX*o*1 by monitoring the activity of the recombinant HsATG4B in the presence of PsTRX*o*1. Before this, we confirmed the redox regulation of the activity of the HsATG4 recombinant protein in the presence of oxidizing H_2_O_2_ and reducing DTT treatments. The activity of the recombinant protein (HsATG4B His-tag of 47 kDa) was demonstrated by the 17 kDa peptide appearing as a result of the ATG8 cleavage (Figure 12A, lane 2) confirming the functionality of the protein when reduced by DTT. In order to determine whether ATG4 activity is influenced by the redox environment, the protein was incubated in the presence of oxidizing (H_2_O_2_) or reducing (DTT) compounds. Pretreatment of HsATG4B with 1 and 3 mM of H_2_O_2_ (Figure 12A, lanes 3 and 4) decreased the processing activity of ATG4 and resulted in the loss of most of the processed peptide, while DTT restored the activity (Figure 12A, lanes 5 and 6). In fact, H_2_O_2_ induced the oligomerization of the protein, while DTT disrupted it, allowing monomerization, which implied that ATG4 is inhibited by oxidation and activated by reduction both being fully reversible. A control experiment included the activity of individual recombinant His-tag proteins ATG4 and ATG8 after the DTT treatment (Figure 12B). These results indicate that the activity is regulated by a redox post-translational modification, as previously described for yeast and *Chlamydomonas* ATG4.

### 3.8. ATG4 Activity Is Redox-Regulated by Thioredoxin o1 In Vitro

**Figure 13:**
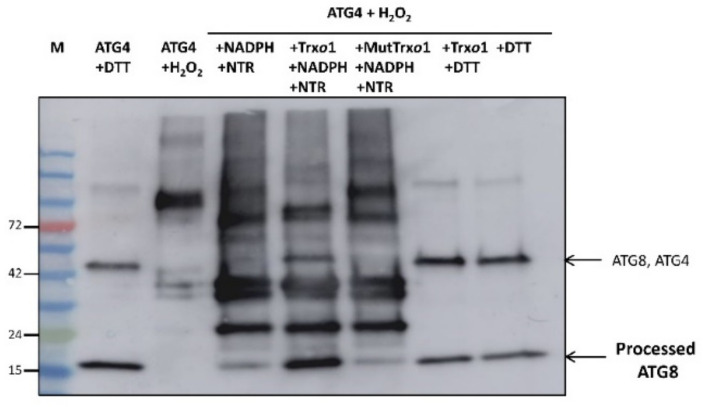
Regulation of ATG4 activity by oxidation and reduction by the TRX*o*1 system. ATG4 activity was monitored by following the cleavage of recombinant quimeric His tag ATG8 substrate from the unprocessed to the processed pATG8 forms (indicated by arrowheads) by Western blot analysis using anti-His antibody. His tag HsATG4B was incubated with 15 µM DTT (lane 2) and 1 mM of H_2_O_2_ (lane 3). After oxidation with H_2_O_2_, the sample was reduced by the NADPH/TR/TRX*o*1 system (lane 5) or NADPH/TR/MutTRX*o*1 (TRX*o*1C37S mutated variant, lane 6). Controls in the absence of TRX*o*1 (lane 4) and reduction by DTT instead of TRX system (lanes 7 and 8) were also run (see material and methods section for details). Marker (M) proteins were loaded in lane 1 for molecular weight position.

In order to test the ability to reduce ATG4 by the TRX system, including TRX*o*1, NADPH, and NTR, we used the recombinant proteins PsTRX*o*1 and AtNTRA, which were previously cloned, expressed in *E. coli,* and purified [26]. Reduced DTT-treated recombinant HsATG4B presented processing activity as seen from the 17 kDa cleavage product (Figure 13 lane 2). This activity decreased when DTT-treated HsATG4B was oxidized by 1 mM H_2_O_2_ (lane 3), which was responsible for the oligomerization, as demonstrated by the appearance of a high-molecular-weight signal in Figure 13. Instead of DTT, we then used the TRX system to reduce H_2_O_2_-treated ATG4B. The activity was monitored with NADPH/NTR (without TRX*o*1) (lane 4), and the activity observed was clearly higher in the presence of TRX*o*1 (lane 5). Very low processing activity, similar to that observed with NADPH/NTR only, was observed when a catalytic cysteine mutant, TRX*o*1C37S (MutTRX*o*1), was used (lane 6). ATG4 activity was lower when TRX+DTT or DTT alone were used as reductants (lanes 7 and 8) instead of NADPH/NTR. These results point to TRX-dependent regulation of ATG4 activity possibly due to the reduction of a regulatory disulfide bond.

## 4. Discussion

In plants, the regulation of autophagy has acquired notable interest due to its favorable response to different stress conditions including oxidative stress, endoplasmic reticulum stress, nutrient deprivation, Cd treatment, drought, salinity, and pathogen response [7,38,39,40,41,42]. In the present work, higher *ATG4* gene expression and protein content of the autophagy markers ATG4, ATG8, and lipidated ATG8-PE proteins as well as high ATG4 activity support the involvement of autophagy process in the response of tobacco BY-2 cells subjected to an H_2_O_2_ treatment when PsTRX*o*1 is overexpressed. In this oxidative situation, these changes in OEX cells may be important as a priming effect, probably influencing the response to a subsequent oxidative stress condition. In this scene, autophagy occurs as is also evident from an accumulation of autolysosomes and ATG8 inmunolocalization. Interestingly, this process correlates with increased cell viability demonstrated in the overexpressing PsTRX*o*1 in TBY-2 cells. Recent studies have shown that posttranslational modifications of some ATG proteins are able to control their activity, such as S-nitrosylation or phosphorylation of ATG4b and ATG1 [43,44] or persulfidation of ATG4 and ATG18a [7,45]. We have explored the role that the PsTRX*o*1 protein may play in the autophagy process by redox regulation of the ATG4 protein involved. In the following sections, we will discuss the main findings evidencing the role of autophagy in response to stress and its involvement in cell viability and redox regulation including the TRX system as a modulator of the activity of target proteins that are key components of the autophagy process. The discussed evidence will help support our findings related to the crosstalk between autophagy, cell viability, and redox signaling via TRX. 

### 4.1. Autophagy, Oxidative Stress and TRX 

Autophagy is slightly activated at a basal level to maintain normal human cellular homeostasis and is under several pathological conditions when this degradative process is induced [46]. In plants, a basal housekeeping level also exists under favorable conditions to eliminate the continuously generated damaged structures or cellular components [47,48]. It is under stress conditions that phenotypes are evident, and growth can be seriously compromised [49]. Hence, studying autophagy under these conditions may provide some clues concerning the relevance of the process in the plant response. Many stresses trigger ROS accumulation, which can induce signaling events involved in the plant response, and a key role of autophagy in the clearance of oxidized proteins has been reported. In this sense, Arabidopsis *atg2* and *atg7* mutants contained more oxidized proteins and were hypersensitive to both salt and osmotic stresses [50]. Other examples of the link between ROS and autophagy are the described induction of this process by salt stress and nutrient starvation, which can be blocked by NADPH oxidase inhibitors [39]. Moreover, AOX-dependent ROS signaling is a key event triggering autophagy as evident from experiments in which the scavenging of H_2_O_2_ in *aox19* mutants induced autophagosome accumulation under drought stress [51]. More directly, autophagy has been reported to be induced by H_2_O_2_ or methyl viologen treatments in the roots of Arabidopsis. Moreover, defective mutants in autophagosome formation in RNAi-*AtATG18a* transgenic plants are more sensitive to oxidative stress, presenting a lower degree of degradation of oxidized proteins as a result of the autophagic process [52]. All these experiments point to the involvement of autophagy in the oxidative stress response. In this context, we previously proposed that one of the PsTRX*o*1 functions in TBY-2 cells is to offer protection against ROS, mainly H_2_O_2_. In fact, a lower content of endogenous H_2_O_2_ and oxidative parameters and a decrease in antioxidants after oxidative treatment were reported in *PsTrxo1* overexpressing cells compared to non-overexpressing cells [28]. In this oxidative situation, autophagy may also occur, so it was analyzed whether TRX could play a role in the possible autophagy induction in response to H_2_O_2_ treatment. The analysis of key autophagy markers revealed that *ATG4* gene expression was downregulated in the control and maintained in OEX-treated cells (Figure 2), pointing to the possible establishment of the autophagy process as a result of overexpression. In this context, scarce information exists about the regulation of the *ATG4* gene expression by redox components via the transcription factors involved. Hence, the reason for the observed effect of overexpression of TRX*o*1 on the increase in the *ATG4* gene expression is unclear, although redox modulation cannot be ruled out. Moreover, in our study, the high and maintained ATG4 protein content was parallel to high ATG4 activity also maintained in H_2_O_2_-treated OEX cells (Figure 3), indicating that sufficient activity was present to sustain autophagy. In this sense, it has been reported that under pathological conditions, the ATG4 processing step is very fast, and only a small amount of ATG4B is enough to sustain the activity in yeast and mammalian cells [53]. On the other hand, an increase in the abundance of ATG8 or ATG8-PE protein levels, like the one we found in our cells, may not reflect a higher autophagic flux due to the fact that blockage of autophagosome formation may also result in the accumulation of these markers [54], so other assays are necessary to estimate this flux and thus ratify the occurrence of the autophagic process. Related to this, and in order to look more closely at this occurrence of autophagy, we monitored autolysosome and autophagosome accumulation using Lysotracker and an anti-ATG8 antibody as markers for autophagy visualization [55,56]. In our study, the accumulation of lytic vesicles was visualized 24 h after oxidative treatment in the OEX cells treated with the inhibitor E-64d, an effect that was reversed in the presence of 3-MA (Figure 5), an inhibitor that has been shown to block starvation-induced autophagy in BY-2 cells thus avoiding autophagosome formation [5,32]. Adding 3-MA clearly inhibited the number of punctate signals, suggesting the presence of autophagic structures in these cells and not in the non-overexpressing ones, which showed no such accumulation at any of the times analyzed (Figure 6 and Figure 9). These results corroborate the early reported effect of E-64d in tobacco cells, which were seen to accumulate autolysosomes outside the central vacuole [57]. In fact, the mode of action of both inhibitors is different: 3-MA has been reported as a selective inhibitor of the early stages of autophagy by inhibiting PI3K activity, which is required for the nucleation and thus the formation of the autophagosome, while E-64 d inhibits autophagy at the later stage mainly by inhibition of papain-like Cys proteases blocking the endocytotic pathway [32,58].

Our immunolocalization study also sustained the existence of autophagic structures due to the observed increase in the content of ATG8 in the H_2_O_2_-treated OEX cells in the presence of the inhibitor (Figure 6). Among ATG proteins, ATG8 has been considered an ideal marker for ascertaining autophagy activity due to the fact that it is incorporated into the membranes of the autophagosomes before being directed to the lytic compartment to be degraded together with the cargo [54]. All the above-mentioned results point to an interesting relationship between oxidative stress and autophagy linked to the overexpression of TRX*o*1. Moreover, this situation is accompanied by an interesting effect on cell survival as discussed in the following section.

### 4.2. Autophagy, Cell Survival and TRX 

The use of autophagy inhibitors allowed us to evidence the link between autophagy, oxidative stress, and cell viability when PsTRX*o*1 was overexpressed. In our TBY-2 cells treated with H_2_O_2_, autophagy was not evident in the first 2 h, at least considering the response to the inhibitor E-64d, which did not significantly affect the viability of any of the lines (Figure S5). However, in an oxidative situation severe enough to provoke the death of most of the cells of the control line 24 h after H_2_O_2_ treatment (only around 20% of cells survived, Figure 4), around 65% of the OEX1 line survived. The fact that the autophagy inhibitor E-64d induced a decrease in OEX1 line viability by up to 33% at this time, parallel to the maintenance of *ATG4* gene expression presented by these cells, may sustain the existence of an autophagic process occurring with an interesting vital role in cell survival. This effect was still observed 48 h after oxidative treatment when the viability was maintained at 54% and decreased to 33% in the presence of the inhibitor, with the decreases again being reverted by 3-MA (Figure S2). Moreover, at a shorter time after the oxidative treatment, at 14 h, when the control line presented higher viability than at 24 h, the autophagy inhibitor E-64d again did not affect cell viability (Figure 8), suggesting that at this time, basal autophagy may be minimal in non-overexpressing cells, a parallel situation to the reported induction of programmed cell death [28]. Related to the effect of 3-MA on viability, and similar to that found in our cells, Takatsuka et al. [32] demonstrated that 3-MA inhibited autophagy in TBY-2 cells although it did not provoke cell death for at least 2 days at 5 mM or 10 mM concentrations (Figure S3), so cell death could not explain the effect of 3-MA on autophagy. In addition, they confirmed that the presence of both E-64c and 3-MA in culture media did not affect cell death as we have also found (Figure 4 and Figure 8). Furthermore, Voitsekhovskaja et al. [36], using TBY-2 cells, observed a lack of effect of 3-MA on viability, although it was able to revert the effect of concanamycin in the degradation of peroxisomes by autophagy. They described the effect of 3-MA on viability at longer durations after inducing autophagy (after 7 days), considering that in the first 6 days, either starvation-induced peroxisome degradation was incompletely blocked or that it was due to another mechanism, different from autophagy. Recently, the contribution of autophagy to cell survival was confirmed in lace plants where a decrease in the cell death rate was observed when autophagy was enhanced with rapamycin, whereas the inhibition of autophagosome formation or blocking of the cargo degradation process had the opposite effect [59]. In this scene, autophagy mediated by TRX could act as a pro-survival mechanism in our cells, as it has been described in situations where recycling of cellular components is necessary, not only for development but mainly under stressful situations [60]. ROS regulation together with autophagy may act as cooperative mechanisms in the response of plants to environmental stresses in order to allow plant survival. Among the actors of ROS regulation, TRX appears to be a key protein in the regulation of the autophagy process, extending its role towards the regulation of cell survival similar to that described in animals. The signaling pathways for activation of the autophagy process, and how its function switches between cell survival and cell death, are just beginning to be elucidated, with several questions still to be solved. The relationship between autophagy and cell survival and the involvement of dysregulation of redox signaling have been identified in animal systems. For example, increasing autophagy by enhancing *ATG8a* expression in neurons of Drosophila increased lifespan [61] and the ubiquitous overexpression of peroxiredoxin dPrx5 in flies subjected to oxidative stress also extended survival [62] while the combined knockdown of dPrx5 and dPrx3 decreased protein thiol levels and the glutathione (GSH) redox state, provoking pro-apoptotic events and shortening lifespan [63]. In plants, where the process has been less studied, an association of autophagy and longevity has been described when autophagy mutants displayed an early leaf senescence phenotype [64]. However, the exact role of autophagy as being pro-death or pro-survival is unclear [65]. Related to this, in our system, the overexpression of PsTRX*o*1 in TBY-2 cells subjected to H_2_O_2_ treatment allowed maintenance of fresh weight of the cultures parallel to a high cell viability and the occurrence of autophagy. In these H_2_O_2_-treated cells, we previously observed increased catalase activity and decreased H_2_O_2_ and NO content parallel to the maintained GSH redox state as well as the level of PrxIIF. All these changes contributed to an observed delay in cell death, compared to control non-overexpressing cells [28]. It is also interesting to point out that the additional involvement of TRX*o*1 in the TBY-2 cell cycle progression, possibly through its link with the proliferating cellular nuclear antigen (PCNA) and GSH. was also reported. In fact, overexpressing *Pstrxo1* cells presented a higher rate of cell proliferation than non-transformed cells, coinciding with upregulation of the target protein PCNA and an effect on cellular GSH content and localization [26]. The described interaction between PCNA and TRX*o*1 to a certain extent could be contributing to the greater viability in these cells. Now, the present work further adds the involvement of autophagy in the response to the oxidative treatment in overexpressing PsTRX*o*1 cells, especially in their increased viability and maintenance of fresh weight indicative of cell proliferation. Interestingly, similar to that described in our present work, a relationship between TRX, autophagy, and cell survival has been reported in human lens epithelial cells under oxidative treatment by H_2_O_2._ In this situation_,_ overexpression of thioredoxin binding protein 2 (TBP-2), which negatively regulates the thioredoxin antioxidative function, promoted cell injury and autophagy, worsening cell viability [66], with TRX then playing a key role as a pro-survival mechanism. In fact, the role for TRX may be to coordinate the response to oxidative stress, probably through the redox regulation of key components of the autophagy process, an aspect discussed in the following section. 

### 4.3. TRX-Dependent Redox Regulation of Autophagy Target Proteins

HsATG4B is a target of PsTRX*o*1 as we demonstrate by dot-blot and co-immunoprecipitation assays using recombinant proteins, which sustained the redox regulation of ATG4 activity. In fact, PsTRX*o*1 but not its mutated version, together with NADPH and NTRA, is able to recover the lost processing activity of oxidized ATG4 on the ATG8 quimeric substrate, as shown in the activity assays (Figure 12; Figure 13). ATG8 was processed not only by recombinant HsATG4B (described as the most-processing isoforms in animals) [67], but also by our TBY-2 cell extracts, which contained the endogenous ATG4 protein (Figure 3). These results confirm the redox regulation of ATG4 activity by the plant TRX system as a result of the reduction of a regulatory disulfide bond. Interestingly, the participation of TRX in autophagy, particularly in ATG4 regulation, has only been demonstrated in *Chlamydomonas* and *Sacharomyces* [21,22], where TRX*h* and Trx1, respectively, efficiently reduced a disulfide bond between two Cys with low redox potential, corroborating the redox regulation of ATG4 by TRX. 

Autophagy must be finely regulated by the cellular environment, and posttranslational modification of target proteins is emerging as a key mechanism of control. Redox regulation of the autophagy process has been described in animal, yeast, and plant systems as previously commented, but information concerning the specific targets and the effects of this regulation is scarce. Among ATGs, increasing interest is focused on the regulation of ATG4 in animal systems due to its involvement in multiple pathological diseases, recently being proposed as a potential anticancer target [68]. ATG4 is the sole protease among ATGs that regulates autophagy through the first very fast and efficient processing step that only needs a low amount of ATG4 [53], and a second deconjugating step on its substrate ATG8. This last activity is used to recycle ATG8 for the next round of the conjugation reaction, but it is also necessary to promote the elongation step of the autophagosome and its fusion to lysosomes as observed in yeast [11]. In our experiments, OEX cells presented a higher ATG4 protein content after the H_2_O_2_ treatment than control cells, but this was not accompanied by a similar increase in the processing activity. This discrepancy may be explained by possible high delipidation activity of the protein (not measured in our work) or by the existence of a regulatory posttranslational modification such as reduction/oxidation that affects the processing activity, as we have demonstrated. In animal cells, during starvation, autophagy is regulated via superoxide production, while the activity of Arabidopsis ATG4a and ATG4b was shown to be reversibly inhibited by oxidation [69]. In yeast, ROS directly regulate the ATG4 protein, thus activating autophagy and nitrosoglutathione treatment of ATG4 and inhibiting the processing activity of the protein [21], implying that the regulation of critical Cys residues may play an important role in the function of this protein. It has been recently reported in Drosophila that oxidation of a key redox-regulatory cysteine residue in Atg4a increased autophagy and interestingly extended lifespan of the flies [70], although the authors described the redox regulation only for the de-lipidation activity of the ATG4 protein, with this then proposed as a potential pro-longevity target. Furthermore, the persulfidation of Arabidopsis ATG4a has been recently reported as regulating the proteolytic activity of this protease [45] indicating a negative regulation of autophagy by sulfide. Thus, one link between ROS and the induction of autophagy may be ATG4, although it does not seem to be the sole protein in the process that underlies oxidative regulation, since not only is the target of rapamycin (mTOR) redox regulated by Trx1 in the heart [71], but ATG3 and ATG7 have also recently been shown to be redox regulated in human embryonic kidney cells [72]. Interestingly, similar to that found in our work, these last authors described that an active cellular reducing system is required to maintain functional autophagy, in this case, through ATG3 and ATG7 redox regulation, maintaining the active thiols at a low level in order to interact with the ATG4 substrate. In this scene, the inhibition of TRX by overexpression of its inhibitor thioredoxin interacting protein (TXNIP) lowered LC3 lipidation through enhancing inhibitory ATG3 and ATG7 catalytic thiol oxidation, again pointing to the key role of the redox environment and TRX in autophagy regulation, as we have evidenced.

## 5. Conclusions

In this work, we have described that PsTrx*o*1 transformation protects TBY-2 cells from exogenous H_2_O_2_, with the over-expressing cells showing higher viability than non-overexpressing cells. The increase in ATG4 and ATG8 proteins as well as the existence of an autophagy flux revealed by lytic autophagic structures and ATG8 accumulation using an autophagy inhibitor in H_2_O_2_-treated OEX cells point to a pro-survival autophagy process that occurs as a response to the oxidative situation. The high ATG4 activity in OEX PsTRX*o*1 cells, together with the in vitro interaction demonstrated between ATG4 and TRX*o*1 proteins, strongly suggest a possible role of TRX in the redox control of the ATG4 protein. In this sense, TRX may be a component of the autophagy process occurring during the response of TBY-2 cells to an oxidative situation, when it probably collaborates with other defense systems in the increased cell survival rate of the over-expressing cells.

## Data Availability

Data is contained within the article or supplementary material.

## Supplementary Materials

The following are available online at https://www.mdpi.com/article/10.3390/antiox10121884/s1, Figure S1: Control experiment for endogenous ATG4 processing activity of over-expressing PsTRX*o*1 (OEX1) TBY-2 line 24 h after 35 mM H_2_O_2_ treatment (treated cells (-T); Figure S2: Cell viability of the *Pstrxo1* transformed TBY-2 line (OEX1) 48 h after 35 mM H_2_O_2_ treatment in the absence and presence of the autophagy inhibitors E-64d and E-64 plus 3-MA; Figure S3; Cell viability of the *Pstrxo1* transformed TBY-2 line (OEX1) 24 h and 48 h after 35 mM H_2_O_2_ treatment in the absence and presence of the autophagy inhibitor 3-MA; Figure S4: Visualization of H_2_O_2_-induced formation of autolysosomes in overexpressing *Pstrxo1* (OEX1) tobacco BY-2 cells 48 h after 35 mM H_2_O_2_ treatment; Figure S5: Cell viability of Control and two overexpressing *Pstrxo1* TBY-2 lines (OEX1 and OEX2) 2 h after 35 mM H_2_O_2_ treatment in the absence and presence of the autophagy inhibitors E-64d and E-64d plus 3-MA; Figure S6: Western blot of the resulting Co-IP between His-HsATG4B and PsTRX*o*1 recombinant proteins revealed with anti-Histidine and then with anti-PsTRXo1 after mild stripping; Table S1: Primers used in the qRT-PCR analysis.
