# Supplementary material for: Autophagy Is Involved in the Viability of Overexpressing Thioredoxin o1 Tobacco BY-2 Cells under Oxidative Conditions
Source: Antioxidants (Basel). 2021 Nov 25;10(12):1884. doi: 10.3390/antiox10121884 (PMC8698322; doi:10.3390/antiox10121884)
Supplement: Supplementary file 1 [file antioxidants-10-01884-s001.zip › antioxidants-1424866-supplementary.pdf]

antioxidants

Supplementary Material: Autophagy Is Involved in the Viability of Overexpressing Thioredoxin *o*1 Tobacco BY-2 Cells under Oxidative Conditions

Sabrina De Brasi-Velasco †, Omar López-Vidal †, María Carmen Martí, Ana Ortiz-Espín, Francisca Sevilla and Ana Jiménez *

Department of Stress Biology and Plant Pathology, CEBAS-CSIC, 30100 Murcia, Spain; sdebrasi@cebas.csic.es (S.D.B.-V.); omalopez17@yahoo.com (O.L.-V.); mcmarti@cebas.csic.es (M.C.M.); anamaria.ortizespin@gmail.com (A.O.-E.); fsevilla@cebas.csic.es (F.S.)
* Correspondence: ajimenez@cebas.csic.es
† These authors contributed equally to this work.

Table S1. Primers used in the qRT-PCR analysis.

Gene	Sequence	Strand
NtATG4f	5′-AAGAGAGAAGAAAVGGGGAATG-3′	Forward
NtATG4r	5′-CCAACGGAACTAAGAAAAGAACAG-3′	Reverse
qACTf	5′-TACAACGAGCTTCGTGTTGC-3′	Forward
qACTr	5′-ACAAGGAAAGGACAGCCTGA-3′	Reverse

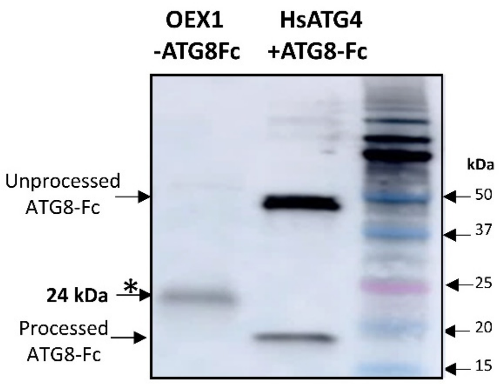

Figure S1. Control experiment for endogenous ATG4 processing activity of over-expressing *Pstrxo1* (OEX1) TBY-2 line 24 h after 35 mM H_2O_2 treatment (treated cells (-T). ATG4 activity of OEX1 cellular extract in the absence of the quimeric substrate His tag ATG8-Fc and positive control of activity of the recombinant HsATG4 monitored by following the cleavage of the substrate from the unprocessed (His ATG8-Fc) to the processed His-ATG8 by western blot analysis using anti-His antibody. Asterisk indicates the non-specific band at 24 kDa. Molecular mass markers (kDa) are indicated.

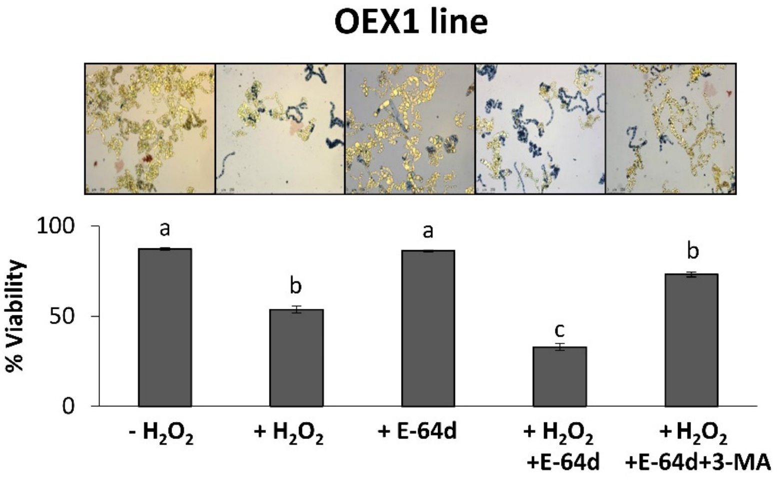

Figure S2. Cell viability of the *Pstrxo1* transformed TBY-2 line (OEX1) 48 h after 35 mM H_2O_2 treatment in the absence and presence of the autophagy inhibitors E-64d and E-64 plus 3-MA. Cell viability (percentage) of overexpressing cells without treatment (-) and after treatment (+) with H_2O_2, E-64d, H_2O_2 + E-64d, H_2O_2 + E-64d + 3-MA was tested using the dye Trypan Blue 48 h after the respective treatments. Values represent mean ± standard error of four independent experiments. Different letters indicate significant differences ($P<0.05$) among treatments according to Tukey's test.

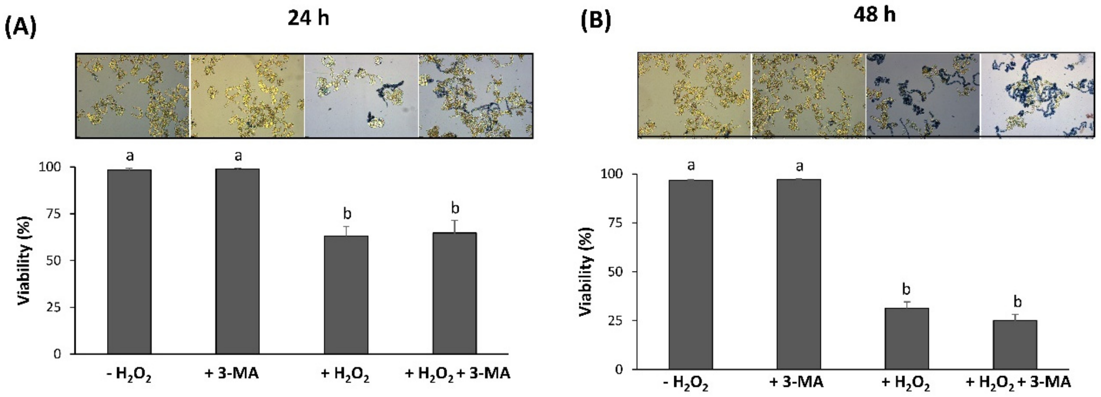

Figure S3. Cell viability of the *Pstrxo1* transformed TBY-2 line (OEX1) 24 h and 48 h after 35 mM H_2O_2 treatment in the absence and presence of the autophagy inhibitor 3-MA. Cell viability (percentage) of overexpressing cells without treatment (-) and after treatment (+) with H_2O_2, 3-MA and H_2O_2 + 3-MA was tested using the dye Trypan Blue 24 h (A) and 48 h (B) after the respective treatments. Values represent mean ± standard error of three independent experiments.

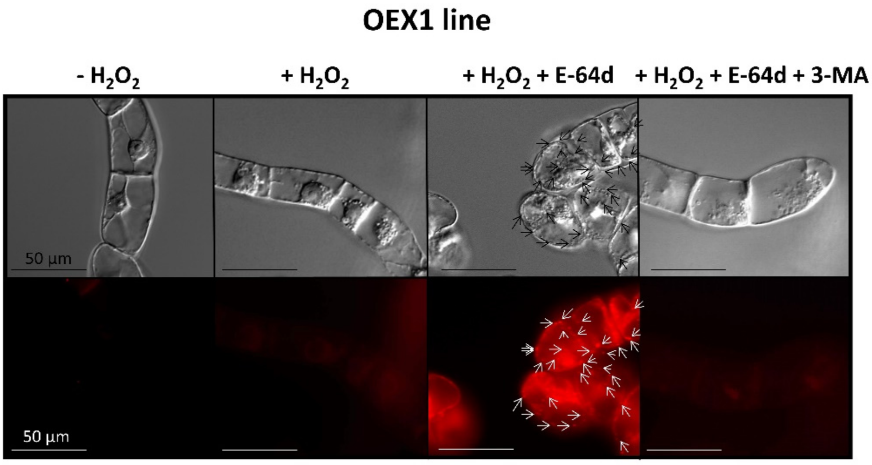

Figure S4. **Visualization of H_2O_2-induced formation of autolysosomes in overexpressing *Pstrxo1* (OEX1) tobacco BY-2 cells 48 h after 35 mM H_2O_2 treatment.** Differential interference contrast (DIC) images and fluorescence images of LDR-dyed structures are shown as representative of three independent experiments. Five day-old TBY-2 cells were treated with 35 mM H_2O_2 alone or in combination with E-64d or E-64d plus 3-MA and observed 48 h after under a fluorescence microscope equipped with a Nomarski optic. Arrows indicate some punctate signals of autolysosomes (red). Scale bars: 50 µm.

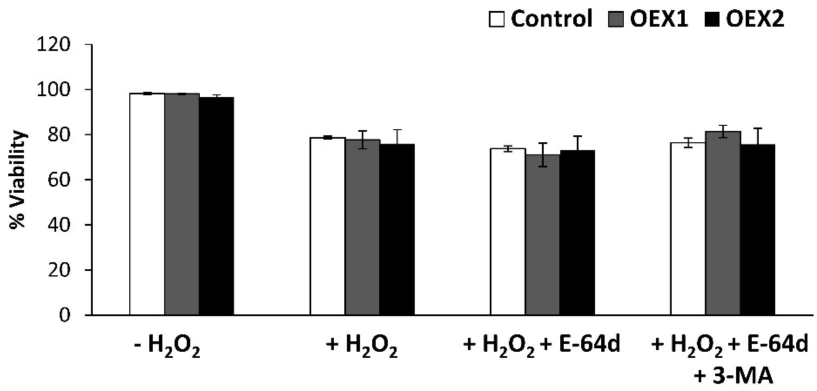

Figure S5. Cell viability of Control and two overexpressing *Pstrxo1* TBY-2 lines (OEX1 and OEX2) 2 h after 35 mM H_2O_2 treatment in the absence and presence of the autophagy inhibitors E-64d and E-64d plus 3-MA. Cell viability (percentage) of cells without treatment (-) and after treatment (+) with H_2O_2, E-64d, H_2O_2 + E-64d, H_2O_2 + E-64d + 3-MA was tested using the dye Trypan Blue 2 h after the respective treatments. Values represent mean ± standard error of four independent experiments.

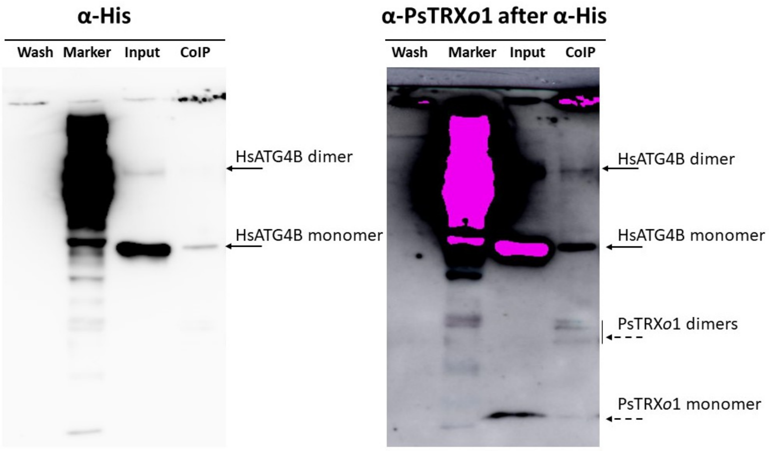

Figure S6. Western blot of the resulting Co-IP between His-HsATG4B and PsTRX*o*1 recombinant proteins revealed with anti-Histidine and then with anti-PsTRX*o*1 after mild stripping. Whole membranes corresponding to the images in Figure 11 (see legend for the description).
